# Comparison of Primary Molar Crown Dimensions with Stainless Steel Crowns in a Sample of Iranian Children

**DOI:** 10.15171/joddd.2014.017

**Published:** 2015-06-10

**Authors:** Hossein Afshar, Arghavan Kamali Sabeti, Mahdi Shahrabi

**Affiliations:** ^1^Associated Professor, Department of Pediatric Dentistry, Tehran University of Medical Sciences, Tehran, Iran; ^2^Assistant Professor, Department of Pediatric Dentistry, Hamedan University of Medical Sciences, Hamedan, Iran; ^3^Associated Professor, Department of Pediatric Dentistry, Tehran University of Medical Sciences, Tehran, Iran

**Keywords:** Dimension, primary molar, stainless steel crown

## Abstract

***Background and aims.*** Due to anatomic variation in tooth anatomy between populations, this study compared the buccolingual (BL) and mesiodistal (MD) dimensions of primary molars with those of stainless steel crowns (SSCs) in anIranian population.

***Materials and methods.*** Impressions were taken from both dental arches of children, and casts were poured. Teeth with caries, restoration, hypoplasia or other dental anomalies were excluded. 216 primary molars were selected and divided into 4 groups of 54 each (maxillary and mandibular first and second primary molars). MD/BL dimensions were measured using a digital caliper with 0.01 mm precision on casts and SCCs (3M brand). Data were assessed using paired t-test, post hoc test and ANOVA. P < 0.05 was considered statistically significant.

***Results.*** The MD dimension of the lower first molar SSC and the BL dimension of the lower second molar SSC had the least difference with the corresponding values of the respective teeth. The MD dimension of the upper second molar SSC and the BL dimension of the upper first molar SSC had the greatest difference with the corresponding values in the respective teeth. Comparison of the two different brands of SSCs for the upper first molar revealed that both types had significant differences with the teeth in terms of both MD (P = 0.0) and BL (P = 0.0) dimensions.

***Conclusion. *** In the studied population, best adaptation was seen in second lower molars and the least adaptationswere seen in first and second upper molars.

## Introduction

Primary teeth play an important role in growth and development of children. Attempts to maintain the primary teeth until the eruption of their permanent successors have resulted in the introduction of many restorative materials and techniques.^1,2^Use of stainless steel crowns (SSCs) is one of these techniques. Humphrey was the first to introduce prefabricated SSCs to pediatric dentistry in 1950.^3^Since then, SSCs have been increasingly used for severely damaged primary teeth. Different SSCs in terms of size, shape and contour (festooned) have been introduced to the dental market by different manufacturers including Denvo Co., Metal Products Co., Rocky Mountain, Unitek SSC and 3M Co.

Selection of an appropriate SSC, in terms of marginal adaptation and proximal fit, has always been challenging for clinicians particularly pedodontists. Previously variations in SSCs and preparation designs were aimed to help with the selection process.^4^The tooth size discrepancy in different populations and ethnic groups is an important issue in this respect that needs to be taken into account. Such differences in some cases are significant.^5-7^ For instance, evidence shows that black Americans have larger teeth than white Americans.^8^ Also, men have larger teeth than women.^9,10^In a research in Iceland on buccolingual and mesiodistal dimensions of primary molar, results showed that they have the largest crown dimensions among European children.^11^ A research in Taiwan was performed to design a suitable SSC. Results showed that their mesiodistal dimension were smaller than Australians and larger than white Americans. The buccolingual dimension were smaller than Indian and Iceland children.^12^Even within one population or ethnic group, crown dimensions can be widely variable. In a study in Spain primary molar dimensions were analyzed. Most difference was seen in first molar.^7^Primary molars have had variations in size and the primary first molar crown has shown the widest variations in dimensions (primary second molar has had the smallest variations).^13^Evaluations of tooth dimensions in different ethnicities and genders just show the differences in tooth size. The question that need to be addressed is if mesiodistal (MD) to buccolingual (BL) ratio of primary teeth in different races is the same and if the available SSC are suitable for different populations. Therefore, this is study was designed to measure the MD and BL dimensions of the primary molar teeth in children presenting to dental care centers in Tehran and to compare these dimensions with the corresponding values in SSCs. At present, pre-trimmed and pre-contoured 3M ESPE SSCs are highly popular. However, a new generation of SSCs has been recently introduced to the dental market. This study focuses on the highly popular 3M SSCs and briefly reviews the recently introduced SSCs.^14^

## Materials and Methods

This cross sectional study was conducted on 283 children aged 4-9 years, referred to the dental care centers in Tehran, Iran. Based on the medical history of children provided by their parents, those suffering from a systemic disease or children with premature birth were excluded from the study. A clinical examination was carried out and the teeth with caries, restorations, hypoplasia or other dental anomalies were excluded from the study. Alginate impressions were made of the remaining 112 children (57 girls and 55 boys). The impressions were poured by dental stone (GC Fuji Rock, EP).Low quality casts were excluded from the study. Of the remaining casts, 107 were selected for the measurements. The selected teeth were divided into four groups of 54 each:

 1. Maxillary first molars, 2. Maxillary second molars, 3. Mandibular first molars, 4. Mandibular second molars

A total of 54 teeth were evaluated in each group (27 teeth belonging to girls and 27 belonging to boys). Also, half the teeth in each group were from the right and the other half from the left quadrants. A digital caliper (Insize, Germany) was calibrated and used to assess the MD and BL dimensions of teeth with 0.01 millimeter precision. The greatest distance between the midpoints of the mesial and distal marginal ridges was measured as the MD dimension of each tooth.

To determine the BL dimension, the greatest BL dimension at the free gingival margin was measured. The MD and BL dimensions of each tooth were measured twice and the mean of the two values was recorded as the final value. For measuring the dimensions of SSCs, numbers 2-7 of Ni-Chro (Ion) SSCs of each tooth in the right and left quadrants (3M ESPE, USA) were selected. The MD dimension of SSCs was measured as in teeth (the greatest distance between the midpoints of the mesial and distal marginal ridges). When placing a SSC, it is optimal to extend the crown margins subgingivally by approximately 1mm.^15-17^For the measurement of BL dimension, we needed to define a line 1mm above the SSC margin in order to match the free gingival margin around teeth. For this purpose, sprue wax with 1mm dimension (used for the fabrication of prosthetic frameworks) was used. The wax was adapted to the SSC margin at the buccal and lingual areas and fixed with super glue. Each SSC was stored at 3°C for 24 hours to allow the wax to set and prevent its deformation. The BL dimension of the SCCs was then measured as the greatest BL dimension at one millimeter above the crown margin. Considering the fact that the thickness of the crown at the cervical margin was reported to be 154µ by Afshar and Mozaffari,^18^twice this amount (0.3 mm) was deducted from the external BL value to obtain the internal BL dimension of SSCs for comparison with the corresponding value in teeth.

A new set of SSCs (MIB, France) have been recently introduced to the dental market. Comparing the occlusal morphology of MIB SSC to 3M SSC of first primary molar shows significant difference. Such this difference is not obvious in visual observation of other SSC. So we decided to measure the SSC of upper first primary molar from MIB brand as an additional assessment.

Despite the assumption that the dimensions of the same size crowns manufactured by the same company are equal, two SSCs of each number (both for right and left crowns) were evaluated to increase the accuracy of the study. The mean value was recorded. Next, dimensions of the teeth were compared to the corresponding values in the respective SSCs.

Since the criterion for the selection of SSCs in practice is the MD dimension of the teeth, the teeth were classified based on this criterion into different SSC sizes. The mean, minimum and maximum differences between the BL and MD dimensions of the teeth and the respective SSCs were calculated. According to these values, the most suitable and unsuitable crowns in terms of MD and BL dimensions were selected.

Data were analyzed by paired t-test, post hoc and ANOVA using SPSS 16.0 software. In this study statistical significance level was set at P <0.05.

## Results

In terms of the MD dimension, the least difference between the teeth dimensions and those of SSCs was seen in the mandibular first molar and the greatest difference was noted in the maxillary second molar (Table 1). For analysis of the relation between SSC, Post Hoc test was used. In mesiodistal dimension significant difference was seen between the SSC of mandibular first molar with, SSC of mandibular second molar, SSC of maxillary first molar and SSC of maxillary second molar (Table 2).

**Table 1:** The mean, minimum and maximum difference between the MD dimension of the teeth and that of SSCs (mm)

**SSCTYPE**	**NUMBER**	**Std. Deviation**	**Mean**	**Minimum**	**Maximum**
**Lower D**	54	0.13639	-0.0491	-0.43	0.15
**Lower E**	54	0.34763	-0.1869	-1.21	0.20
**Upper D**	54	0.32060	-0.2159	-1.05	0.27
**Upper E**	54	0.41188	-0.2493	-1.67	0.23
**Total**	216	0.32765	-0.1753	-1.67	0.27

**Table 2:** Post Hoc analysis with CI of 95% for comparison of different SSC in their mesiodistal difference with teeth

**Main SSC**	**Alternative SSC**	**Mean Difference**	**Std. Error**	**Sig.**
**Lower D**	lower E	0.1378	0.05082	0.050
	upper D	0.1669	0.04741	0.005
	Upper E	0.2002	0.05904	0.007
**Lower E**	lower D	-0.1378	0.05082	0.050
	upper D	0.0291	0.06435	0.998
	Upper E	0.0624	0.07334	0.952
**Upper D**	lower D	-0.1669	0.04741	0.005
	lower E	-0.0291	0.06435	0.998
	Upper E	0.0333	0.07103	0.998
**Upper E**	lower D	-0.2002	0.05904	0.007
	lower E	-0.0624	0.07334	0.952
	upper D	-0.0333	0.07103	0.998

In comparison of the BL dimension, the least difference between the SSCs and the teeth was seen in the mandibular second molar while the maximum difference was seen in the maxillary first molar (Table 3). In buccolingual dimension significant difference was seen between SSC of mandibular second molar, with SSC of maxillary first molar and SSC of mandibular first molar (Table 4).

**Table 3:** The mean, minimum and maximum difference between the BL dimension of the teeth and that of SSCs (mm)

**SSC Type**	**NUMBER**	**Mean**	**Std. Deviation**	**Minimum**	**Maximum**
**lower D**	54	0.6367	0.46805	-0.49	1.64
**lower E**	54	0.1800	0.51094	-0.81	1.34
**Upper D**	54	1.0930	0.43674	0.32	1.85
**Upper E**	54	0.2196	0.57058	-1.23	1.59
**Total**	216	0.5323	0.61891	-1.23	1.85

**Table 4:** Post Hoc analysis with CI of 95% for comparisonof different SSC in their buccolingual difference with teeth

**Main SSC**	**Alternative SSC**	**Mean Difference**	**Std. Error**	**Sig.**
**Lower D**	lower E	0.4567	0.09429	0.000
	upper D	-0.4563	0.08712	0.000
	Upper E	0.4170	0.10043	0.000
**Lower E**	lower D	-0.4567	0.09429	0.000
	upper D	-0.9130	0.09147	0.000
	Upper E	-0.0396	0.10423	0.999
**Upper D**	lower D	0.4563	0.08712	0.000
	lower E	0.9130	0.09147	0.000
	Upper E	0.8733	0.09778	0.000
**Upper E**	lower D	-0.4170	0.10043	0.000
	lower E	0.0396	0.10423	0.999
	upper D	-0.8733	0.09778	0.000

Comparison of the two different brands of SSCs for the maxillary first molar revealed that both types had significant differences with the teeth in terms of both MD and BL dimensions (P = 0.0 and P = 0.0). However, the mean values demonstrated that the recently introduced SSC (MIB) had smaller differences with the teeth in terms of BL and MD dimensions compared to 3M SSCs (Table 5).

**Table 5:** The mean difference between the teeth and the two types of SSCs in terms of MD and BL dimensions using paired t-test

**SSC**	**Number**	**Mean DIFF.**	**Std. Deviation**	**Sig .(2-tailed)**
**(3M) MD**	54	-.2159	.32060	.000
**(MIB )MD**	54	-.0735	.18788	.001
**(3M) BL**	54	.0930	.43674	.00
**(MIB ) BL**	54	.4211	.57353	.002

## Discussion

Since the introduction of SSCs by Humphrey in 1950, they have been extensively used for the restoration of primary and permanent posterior teeth. No other restoration has the ease of use, durability and reliability of these full coverage crowns for the primary teeth.^14,19^The 3M ESPE Company manufactures one of the most commonly used SSCs for the market. These crowns are available in different sizes for the primary posterior teeth. Since it seems that the dimensions of these crowns have been determined based on the epidemiologic data of the manufacturing country. The important question here is whether these crowns are suitable for use in patients of other populations (in terms of teeth dimensions). In order to answer this question, we measured the buccolingual (BL) and mesiodistal (MD) dimensions of SSCs and compared them with the corresponding values in the respective teeth.

The MD dimension of the SSC of the mandibular first molar had the greatest adaptation and the least difference with the corresponding value in the respective teeth. This SSC ranked third in terms of the adaptation of its BL dimension to that of teeth. Such differences indicate that when placing a SSC for the mandibular first molar, the SSC with an ideal MD adaptation should be chosen and then buccal and lingual surfaces of the tooth at the cervical area must be prepared. Considering the presence of mesiobuccal bulge of this tooth, the preparation is usually done at this area.

The SSC of the mandibular second molar ranked second in terms of MD adaptation. However, this SSC had the greatest BL adaptation to the teeth. This finding indicates that if this crown fits the tooth mesiodistally, buccal and lingual surfaces of the tooth require minimum preparation at the cervical area. It is expected that the selected SSCs fit well with the teeth that have received a standard and classic preparation.

On the other hand, the SSC of the maxillary first molar ranked third in terms of MD adaptation. The BL dimension of this SSC had the least adaptation with that of the respective teeth. In other words, a mesiodistally ideal SSC was too small buccolingually for these teeth and requires extensive preparations of buccal and lingual surfaces in order to fit well. If a larger size SSC is selected (requiring less preparation), we will encounter space shortage mesiodistally; which will lead to incomplete seating of the SSC and its subsequent rotation in most cases. Such misfit is partly due to the presence of mesiobuccal bulge (similar to the mandibular first molar). By preparation (reduction) of this area, the BL dimension of the tooth is decreased to some extent. However, the difference between the BL dimension of this tooth and the respective 3M SSC is considerably high and thus, reduction of the cervical ridge prominence does not seem to provide an ideal fit. Therefore, a theory suggests that the morphology and the BL and MD dimensions of the maxillary first primary molars in Iranian population may be different from those of the target population of 3M SSCs. The MIB SSCs are manufactured in totally different designs with different MD and BL dimensions particularly for the upper D; which confirms this theory. Such observations indicate that the discrepancy between the dimensions of the 3M SSCs and those of the teeth in different populations has been probably so obvious that the MIB company decided to manufacture SSCs of different MD and BL dimensions. Using MIB SSCs in the upper D perfectly solves this problem and provides an ideal fit with minimum preparation in most cases.

The SSC of the upper E ranked fourth in terms of MD adaptation and ranked second in terms of BL adaptation. If adequate attention is not paid, such misfit in the MD dimension can cause the over-contour in distal and lead to ectopic eruption of the permanent first molar tooth .^20^If a smaller size SSC is selected to achieve MD fit, the buccal and lingual surfaces will require excess preparations above the standard and classic limit.

Variations in teeth size in different races do not usually pose a significant problem considering that SSCs are manufactured in different sizes. But, since SSCs are selected based on their ideal MD adaptation to the teeth (to achieve appropriate contact with the adjacent teeth), the important issue is that whether the selected SSC has adequate BL adaptation to the tooth particularly at the cervical area, where is the most important site for achieving a perfect fit.

Since it is almost impossible to change the MD dimensions of SSCs and since it is much easier to change the SSC margins (circumference), the required changes are usually made at the circumference of the SSCs. Crimping is among such modifications ^21^that decreases the circumference of the crown. One study showed that this technique decreased the circumference by 7%.^18^Another technique is to cut the SSC margins if they are too long for the tooth. This positively changes the circumference of the SSC.^22^Making a cut in the buccal or lingual surface of the crown and soldering the two ends at a new position with greater adaptation to the tooth surface is another technique that also decreases the SSC circumference. To increase the circumference, a piece of band can be soldered to the incision site in the buccal or lingual surface of the crown.^23^The latter two techniques are time consuming and associated with some problems such as inadequate fit of the margins at the marked points, the need for more polishing at the margins and leakage at the soldered site.^24,25^However, if in any population, the discrepancy between the MD dimension to circumference ratio of teeth and SSC (either positive or negative) is too large to be compensated by the mentioned adjustments, such discrepancy must first be confirmed by epidemiologic studies and then reported to the manufacturing company. The manufacturing companies are required to take into account the variations in these ratios in different populations before the production of SSCs. By doing so, the need for excess preparations is obviated and time is saved; which is particularly important in young, uncooperative children.

## Conclusion

The BL and MD dimensions of the primary molars were different from the corresponding values in SSCs. Such differences were greater for the maxillary first and second molars. The newly introduced SSCs for the maxillary first molars by the MIB Company had superior adaptation to the teeth, and replacing the 3M SSCs with the MIB crowns has advantages such as saving time and more preservations of tooth structure.
